# Measurement of Extraneous and Germane Cognitive Load in the Mathematics Addition Task: An Event-Related Potential Study

**DOI:** 10.3390/brainsci12081036

**Published:** 2022-08-04

**Authors:** Chao-Chih Wang, Peter Kuan-Hao Cheng, Tzu-Hua Wang

**Affiliations:** 1 Research Center for Education and Mind Sciences, National Tsing Hua University, Hsinchu 300193, Taiwan; 2 School of Education Sciences, Huizhou University, Huizhou 516007, China; 3 Center for Research in Cognitive Sciences, National Chung Cheng University, Chiayi 621301, Taiwan; 4 Department of Education and Learning Technology, National Tsing Hua University, Hsinchu 300193, Taiwan

**Keywords:** brain activity, cognitive load, mathematics computation, mathematics education, event-related potential

## Abstract

Cognitive load significantly influences learning effectiveness. All the three types of cognitive load—intrinsic, extraneous, and germane—are important for guiding teachers in preparing effective instructional designs for students. However, the techniques used to assess the relationship between brain activity and cognitive load during learning activities require further investigation. This study preliminarily examined cognitive load during mathematics computations based on cognitive-load theory. We used event-related potentials to compare carryover and without carryover additions under three types of stimuli (uncoloured Arabic numerals, colourful Arabic numerals, and Chinese numerals) to measure learners’ cognitive load. According to the concept and rationale of cognitive-load theory, the design defined the extraneous and germane cognitive load to measure the N1 and P2 components and the relevant behavioural data. The highest P2 amplitude was observed in the Chinese numerals condition as extraneous cognitive load, and the N1 component was observed in the colourful Arabic numerals condition as germane cognitive load. Thus, both components may play an important role in extraneous and germane cognitive load. Additionally, these exhibit negative correlations during mathematical computations. This study’s findings and implications offer insights into future ways for assessing cognitive load using brain imaging techniques and potential applications for brain–computer interfaces.

## 1. Introduction

In the field of education, the relationship between instructional environment design and learning effectiveness has always been a crucial concern; this relationship has been explained by cognitive-load theory [1]. According to this theory, long-term memory and working memory are connected to each other through limited capacity [2,3]. The main function of working memory is to process new information obtained by attention and recognition, organise, contrast, compare, or understand the information, and pass it on to long-term memory, which then adds meaning to the new information, organises, and stores it for subsequent use [3]. Kirschner pointed out that working memory can process new information regarding only seven items or elements at a time. Additionally, because this area organises, controls, compares, or understands new information, only two or three items or elements of that new information can be processed at one time [4,5]. This indicates that learners arrange their cognitive resources during learning activities, and the instructional format is often the main external cause of cognitive overload, which may negatively affect teaching and learning benefits. In other words, if we reduce the size of the external source of the cognitive load, we can create more space for learning, leading to better learning and transfer [6].

According to cognitive-load theory, there are three main types of cognitive load: intrinsic, extraneous, and germane [7]. Bannert presented a detailed discussion of the application of cognitive-load theory in education, concluding that intrinsic cognitive load mainly develops according to the nature of teaching materials, such as the level of difficulty and relevance of the teaching material contents, whereas extraneous cognitive load mainly develops from the methods of presenting the teaching materials [6]. Extraneous cognitive load is not helpful for learning, mainly because of the poor design of teaching materials and activities. This can increase the requirement of working memory and adversely affect learning effectiveness. The third type, germane cognitive load, also develops because of instructional design and teaching material presentations [3,6]. However, this type of cognitive load is meaningful for learning because it encourages learners to use spare working memory areas to conduct in-depth construction and automation of schemas [6]. 

By applying cognitive-load theory to education, researchers have emphasised the capacity limitation of learners’ cognitive load in the context of development and design of teaching activities and materials, thereby improving learning effectiveness [1,3,6,8]. In *Nine Ways to Reduce Cognitive Load in Multimedia Learning*, Mayer and Moreno proposed nine approaches to reduce the cognitive load of learners; these approaches are to be implemented while designing a multimedia learning environment [9]. Mayer and Moreno posited that the total cognitive processing intended for learning comprises essential processing, incidental processing, and representational holding. Essential processing refers to the cognitive processes required to make sense of the presented material, including the selection and organisation of words and images and their integration [9]. Incidental processing relates to cognitive processes that are not required to make sense of the presented material but are primed by the design of a learning task [9]. Representational holding includes cognitive processes aimed at holding mental representations in the working memory [9]. Additionally, Mayer and Moreno noted that once the extent of total cognitive processing exceeds the learners’ cognitive capacity, it affects their learning effectiveness; their nine approaches to reducing cognitive load are based on the aforementioned hypothesis. Subsequently, Mayer further collated relevant literature and proposed that the cognitive processes of selection, organisation, and integration should be considered when using multimedia for designing new learning materials, and that the overload of visual and verbal channels in working memory should also be noted during the learning process [10]. Among them, ‘reducing extraneous processing’ means avoiding cognitive operation that is not directly related to learning objectives, ‘managing essential processing’ means avoiding necessary cognitive operation exceeding learners’ cognitive capacity, and ‘fostering general processing’ means improving learning motivation and applying the available cognitive capacity to promote the understanding of learning materials [8]. The importance of cognitive capacity in learners’ learning is emphasised upon. According to the arguments raised by Mayer and Moreno [9], Mayer [10], Bannert [6], Kirschner [3], Sweller [1], and Sweller, van Merrienboer, and Paas [7], if the cognitive load developed by instructional design and teaching-material presentations exceeds learners’ cognitive capacity, it negatively affects their learning effectiveness. 

Pass et al. stated that methods for measuring learners’ cognitive load can be divided into three categories: subjective, physical, and task- and performance-based techniques [11]. Pass mainly represented subjective techniques using a rating scale to allow participants to self-report the mental effort they expended [12]. Pass et al. stated that this self-rating method is often used in research, but its measurement results are often questioned [11]. Physical techniques include the heart rate variability test proposed by Paas and van Merriënboer [13] and the eye activity test proposed by Beatty and Lucero-Wagoner [14] and Van Gerven, Paas, van Merriënboer, and Schmidt [15]. According to Pass et al. [11], the task-evoked pupillary response activities proposed by Beatty and Lucero-Wagoner [14], including mean pupil division, peak division, and latency to peak, are of high value for the measurement of cognitive load; however, the measurement of cognitive load using heart rate variability tests is of little value. Studies by Sweller [1] and Brünken, Plass, and Leutner [16] are representative of task- and performance-based techniques, which are divided into primary and secondary task measurements. Primary task measurement refers to the participants’ performance in task execution, and secondary task measurement refers to the primary task performance when executing a secondary task. Secondary task performance reflects the cognitive load developed by the primary task, and its measurements include reaction time, accuracy, and error rate. Pass et al. stated that although task- and performance-based techniques are valuable for measuring cognitive load, researchers rarely use them, mainly because secondary task performance may be affected by primary task design, thus affecting research results. 

According to Pass et al. [11] and Pass, Ayres, and Pachman [17], subjective techniques are the most commonly used to measure cognitive load. Moreover, eye activity under physical techniques is useful for measuring cognitive load. Although task- and performance-based techniques are suitable for measuring cognitive load, the interference between task design and tasks affects measurement results. Pass et al. [11] reported that brain activity is another physiological indicator suitable for measuring cognitive load. According to Pass, Ayres, and Pachman [17], functional magnetic resonance imaging (fMRI) can also be used to measure cognitive load, as it has been used to dissociate the verbal, spatial, and central executive units of working memory and to identify physical capacity constraints in working memory [17,18,19,20]. 

Since cognitive load markedly influences learning effectiveness, and based on the suggestions of Pass et al. [11] and Pass et al. [17], more cognitive-load measurement techniques should be developed using physiological elements. This study explored the application of event-related potentials (ERPs) to measure cognitive load by examining brain activity. Some studies have examined cognitive-load theory using the electroencephalogram (EEG) technique [21,22,23,24,25,26,27]; however, they were hampered by limitations such as noise, which easily affects EEG results and participants’ heartbeat and breathing rates [28].

In contrast to EEG activity, ERP is more specific because it is more direct in measuring specific components of cognitive function [29]; EEG data may not directly measure cognitive function signals. Therefore, the present study assessed cognitive load based on cognitive-load theory using ERP. Moreover, the experiment primarily demonstrated extraneous and germane types of cognitive load in relation to ERP components as a measurement technique.

In the present study, we attempted to measure two types of cognitive load: extraneous and germane. However, there remains the issue regarding the rationale for these two conditions. Therefore, we defined extraneous and germane conditions based on cognitive-load theory [3,6,7]. Hence, this is a preliminary study examining the relationship between cognitive-load theory and mathematical computation. The experimental design involved addition operations, with and without carryover, using uncoloured Arabic numerals, colourful Arabic numerals, and Chinese numerals. The study participants were asked to calculate the items using addition without carryover (e.g., 1 + 2 = 3) and with carryover (e.g., 9 + 2 = 11). The numbers and symbols were presented sequentially for the participants in each trial. They judged the accuracy of the additions. As for the measurement of extraneous cognitive load [7], the extraneous condition defined that it is not appropriate to access information and spend more cognitive load on inefficient learning or solving problems. Therefore, Chinese numerals were adopted. These are similar to words and are rarely used in Taiwan because it is not convenient to calculate, as Americans use words (e.g., nine plus two equal eleven) to calculate. In special situations, traditional Chinese numerals are widely employed in finance, primarily for writing sums on checks, so that financial settings cannot be altered by forgery. A mathematical operation involving Chinese numerals was made more difficult by the requirement that participants convert the Chinese characters to standard Arabic numerals. Chinese numerals are unfamiliar for participants and belong to low-frequency words. According to some studies [30], there may be a large P2 for accessing Chinese numerals. In this condition, the early phase of translation would result in a high cognitive load among the participants but would not indicate their overall performance. As for germane cognitive load [3,6,7], the present study found that participants spent more effort and accessed this kind of information more efficiently. Given the definition, the colourful Arabic numerals were selected because coloured words may enhance performances [31,32]. Although colourful numbers may induce more cognitive load for participants, there are different colours to process, and hence, may enhance performance of germane cognitive load. This number presentation strategy shows that although it adds colour information unrelated to mathematical computation, it may help participants focus on the information required for answering. In the uncoloured Arabic numerals condition, uncoloured numbers were shown to the participants as a control condition.

## 2. Materials and Methods

### 2.1. Participants and Design

Thirty-two university students (18 women and 14 men; age range: 20–31 years, *M* = 22.24 years, *SD* = 2.24 years) participated in this study. All participants were native Mandarin speakers and right-handed, with normal or corrected-to-normal vision. Informed consent was obtained from all participants before the EEG experiment. Each participant received a payment equivalent to 17 USD (500 TWD) for their contribution to the experiment. This study was approved by the Ethics Committee of the National Tsing Hua University (10711HT076). As previously stated, the experimental design included the extent of difficulty arising from the addition operations with or without carryover and the complexity of material types, which represents a cognitive-load level in information processing and maintenance. Two factors involving carryover and three types of the materials were manipulated and collected reaction time and accuracy for behavioural data, and N1, P2 for ERP data. Three female participants were removed from the subsequent analysis because of corrupt and irretrievable EEG raw data in one of the three conditions. 

### 2.2. Materials

**Figure 1:**
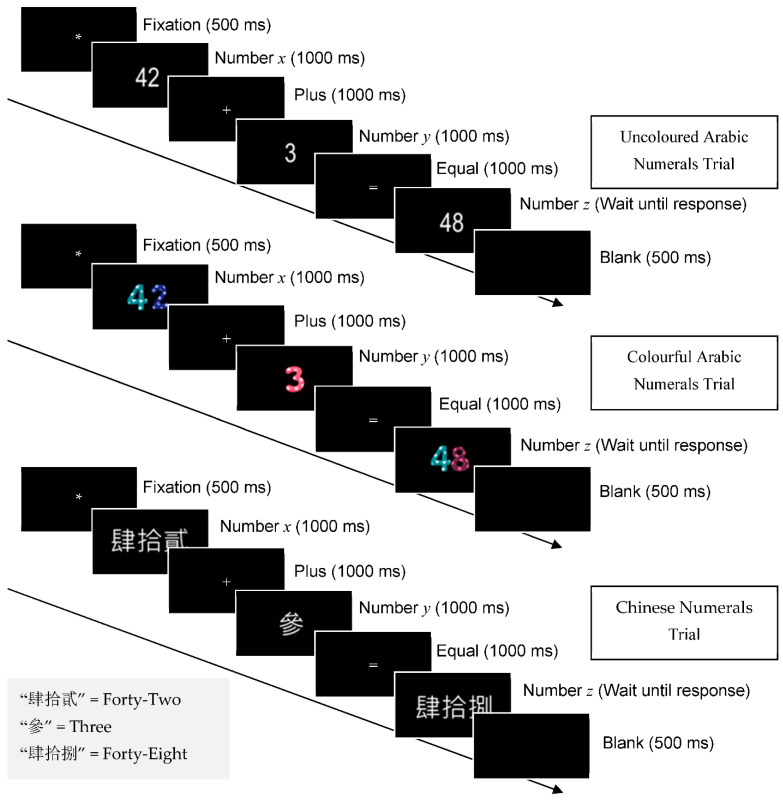
Schematic representations of the conditionwise trials and their respective timings for the uncoloured Arabic numerals trial (**top**), colourful Arabic numerals trial (**middle**), and Chinese numerals trial (**bottom**). See the text for further details.

For the basic arithmetic task of addition, we employed three types of material: uncoloured Arabic numerals, colourful Arabic numerals, and Chinese numerals (Figure 1). Both the first two types use the Arabic numeral system (one to ten numerical digits: 1, 2, 3, 4, 5, 6, 7, 8, 9, and 10) to represent the number of stimuli. In the colourful Arabic numerals stimuli, each number digit had specific colour content, and this was kept the same throughout all the trials. The Chinese numerals were adopted from Chinese characters, which are extensively used in finance; each number digit has a corresponding Chinese character to represent it (one to ten Chinese characters: 壹, 貳, 參, 肆, 伍, 陸, 柒, 捌, 玖, and 拾). The augend (X number) ranged from 1 to 91, the addend (Y number) ranged from 4 to 9, and the sum (Z number) ranged from 4 to 94 (e.g., 1 + 3 = 4, 87 + 7 = 94). The experiment was run on a desktop computer; the viewing distance was maintained at 70 cm. The visual angle of each stimulus in the normal and colourful conditions was approximately 2.05° horizontally and 1.41° vertically. In the Chinese character condition, it was 4.91° horizontally and 1.23° vertically. 

### 2.3. Procedure

We instructed the participants to solve the simple addition task in an acoustically shielded cubicle and collected their EEG data separately for each condition while performing the tasks. In this study, there were three separate EEG acquisition sessions (uncoloured Arabic numerals, colourful Arabic numerals, and Chinese numerals). The participants were divided into six groups, and the presentation order of the three conditions was counterbalanced (ABC, ACB, BAC, BCA, CAB, and CBA). Each session consisted of 80 trials that lasted for less than 10 min.

As illustrated in Figure 1, each trial started with a white asterisk (*) appearing in the centre of a black screen for 500 ms, indicating the beginning of the task. After the asterisk disappeared, a number (*x*) (e.g., the Arabic numeral ‘42′ and Chinese numeral ‘肆拾貳’) appeared in the centre of the screen for 1000 ms. The participants were asked to keep this number in mind for the subsequent arithmetic operation. Next, the ‘+’ sign appeared for 1000 ms, and another number (*y*) (e.g., the Arabic numeral ‘3’ and Chinese numeral ‘參’) was presented right behind the plus sign for 1000 ms. The participants were asked to mentally add the number x and number y (e.g., 42 + 3). The ‘=’ symbol was then displayed on the screen for 1000 ms before being followed by a number (*z*) (e.g., the Arabic numeral ‘48′ and Chinese numeral ‘肆拾捌’) to verify if the participants’ sum of (x + y) equalled **z**. If the equation was true, the participants were asked to press the slash key with their right hand; otherwise, they used their left hand to press the ‘z’ key (for example, in Figure 1, 42+3≠48, hence, the participants should press the ‘z’ key). A blank screen was shown for 500 ms between trials. 

The simple addition task without carryover (e.g., 23 + 4 = 27) was used in half of the trials, whereas the addition task with carryover (e.g., 16 + 8 = 24) was used in the other half. Under each session, half of the equations were true (e.g., 92 + 3 = 95), and the remaining half were false (e.g., 76 + 5 = 91). The presentation order of the simple addition tasks with and without arithmetic operations was randomly determined by the E-Prime 3 programme. The participants took approximately 45 min to complete the experiment, and they were allowed to take a break at their own pace between the EEG acquisition sessions.

#### 2.3.1. ERP Recording

EEG activity was recorded using a 32-channel Quik-Cap and amplified with a SynAmps 2 system (Compumedics Neuroscan; Charlotte, NC, USA). The ground electrode was placed on the mid-frontal scalp, and all channels were referred to the left (M1) side of the mastoid. The montage included 6 midline sites (FZ, FCZ, CZ, CPZ, PZ, and OZ) and 12 sites over each hemisphere (Fp1/Fp2, F3/F4, F7/F8, FC3/FC4, FT7/FT8, C3/C4, T3/T4, CP3/CP4, TP7/TP8, P3/P4, T5/T6, and O1/O2). Additional electrodes were employed to monitor ocular artefacts. Electrodes above and below the left eye were used to record the vertical electrooculogram (EOG). The horizontal EOG was recorded from the outer canthus of each eye. The impedance was maintained at or below 5 K before beginning the EEQ acquisition. At each electrode location, continuous EEG activity was sampled at 1000 Hz, amplified with SynAmps 2 AC amplifiers, and digitised at a 24-bit resolution.

#### 2.3.2. ERP Analyses

We used the EEGLAB toolbox [33] to preprocess the data and ensure data quality. All channels were first filtered using a 0.05–50 Hz band-pass filter and then using the ‘runica’ independent component analysis (ICA) algorithm to identify and minimise eye movements and blink artefacts. After ICA, all EEG channels were re-referenced to the linked (M1 + M2)/2 mastoid regions using the ERPLAB plug-in toolbox [34]. The continuous EEG was then segmented from −100 to 1000 ms relative to the onset of the number of stimuli, which included a 100 ms prestimulus baseline period. Epochs with excessive muscle or body movement noise (100 μV) in any of the 32 channels were automatically rejected by using the moving-window peak-to-peak threshold. At least 30 trials were accepted for each condition and used for further group analysis. For each participant, the remaining epochs were averaged separately according to the EEG acquisition sessions in each condition at three different levels: number X came from both addition trials, number (y) came from addition without carryover trials, and number Y came from addition with carryover trials. The mean amplitudes of ERPs were quantified over three time periods: N1 (100–150 ms), P2 (200–300 ms), and the late positive component (LPC) (600–900 ms). An ANOVA was initially conducted for each period from the FZ channel. When significant effects involving the conditions of interest were observed, subsidiary ANOVAs or t-tests were performed for pairwise comparisons.

We examined the ERP components that reflect covert cognitive-load adjustment and noted load patterns during high- and low-workload situations. The following analyses were performed for all the hypotheses. First, if the difference in ERP patterns evoked by the different material types of numbers reflected a cognitive-load level in information processing and maintenance, the N1, P2, and LPC components would be evoked by the first number X but with distinct mean amplitudes of different stimuli because of the complex loading of visual information. Second, since the arithmetic operation actually began with the second number Y, the difficulty of cognitive load resulting from the addition without carryover and addition with carryover conditions might induce more complex components in N1, P2, and LPC compared to those induced by the number X. Finally, if N1 and P2 ERP components are indices of cognitive load during the mathematical operations, there would be high N1 observed in the colourful Arabic condition and high P2 in the Chinese numerals condition.

## 3. Results

### 3.1. Analysis of Behavioural Data

Table 1 presents the mean rates of ‘Yes’ responses to each type of arithmetic task (including hit and false alarm) and corrected performance (hit–false alarm), rectified for guessing. The reaction time (RT) was the average values of the response time of correct hit performance. In each condition, trials with reaction time exceeding by 2.5 standard deviations from the mean were excluded as outlier responses. To examine the effects of material category and difficulty, a 3 (uncoloured Arabic numerals, colourful Arabic numerals, Chinese numerals) × 2 (without carryover vs. carryover) × 2 (female, male) mixed design ANOVA was performed on the corrected behaviour performance (hit rates–false alarm rates). The main effect of material category was found to be significant [*F*_(2, 54)_ = 4.393, *p* = 0.017, $\eta_{p}^{2}$ = 0.140]. Post hoc analysis revealed that the corrected behaviour performance during the Chinese numerals condition (*M* = 0.91, *SD* = 0.08) was significantly lower than that under the colourful Arabic numerals condition (*M* = 0.95, *SD* = 0.05) [*t*_(28)_ = 2.787, *p* = 0.009, *d* = 0.600]. The main effect of difficulty was found to have the following values: [*F*_(1, 27)_ = 16.672, *p* < 0.001, $\eta_{p}^{2}$ = 0.382], indicating that easier addition without carryover tasks (M = 0.96, SD = 0.04) allowed a higher corrected performance than difficult additions with carryover tasks (*M* = 0.91, *SD* = 0.07) [*t*_(28)_ = 4.140, *p* < 0.001, *d* = 0.877]. The main effect of sex on corrected behaviour performance was not significant [*F*_(1, 27)_ = 0.197, *p* = 0.661, $\eta_{p}^{2}$ = 0.007] and neither on the remaining interactions.

Regarding the hit response RT, the same three-way mixed design ANOVA indicated that the main effect of the material category also reached significance [*F*_(2, 54)_ = 54.151, *p* < 0.001, $\eta_{p}^{2}$ = 0.667]. The post hoc analysis revealed that in the uncoloured Arabic numerals condition (*M* = 515, *SD* = 118), hit RT was shorter than in the colourful Arabic numerals condition (*M* = 567, *SD* = 107) [*t*_(28)_ = 3.867, *p* < 0.001, *d* = 0.462] and the Chinese numerals condition (*M* = 719, *SD* = 139) [*t*_(28)_ = 8.805, *p* < 0.001, *d* = 1.582]. The RT in colourful Arabic numerals was also shorter than in the Chinese numerals condition [*t*_(28)_ = 6.068, *p* < 0.001, *d* = 1.225]. The main effect of difficulty was also revealed to be significant [*F*_(1, 28)_ = 25.012, *p* < 0.001, $\eta_{p}^{2}$ = 0.481], which was driven by the addition without carryover wherein the RT was faster (*M* = 582, *SD* = 98) than to additions with carryover (*M* = 618, *SD* = 111) [*t*_(28)_ = 5.098, *p* < 0.001, *d* = 0.344]. However, there was no significant main effect of sex on RT [*F*_(1, 27)_ = 1.663, *p* = 0.208, $\eta_{p}^{2}$ = 0.058].

**Figure 2:**
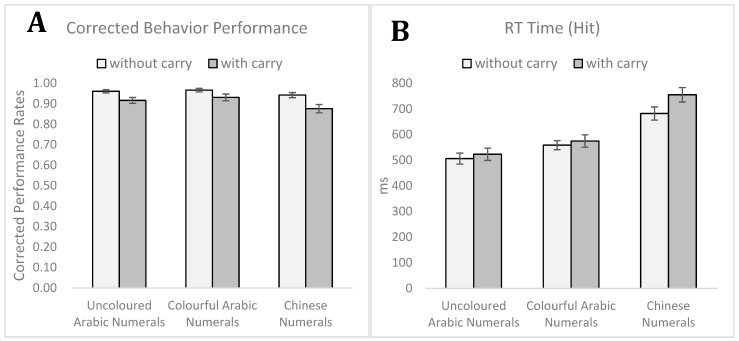
The mean corrected behavioural performance in percentages (hit–false alarm) (**A**) and mean reaction time in milliseconds (hit response) (**B**) of three conditions in the simple addition without carryover and with carryover arithmetic operation. Error bars indicate standard errors.

The interaction between material category and difficulty was also significant [*F*_(2, 56)_ = 10.353, *p* < 0.001, $\eta_{p}^{2}$ = 0.270]. Further analysis revealed that the effect of material category was significant in the additions without carryover [*F*_(2.56)_ = 42.036, *p* < 0.001, $\eta_{p}^{2}$ = 0.600], and post hoc analysis revealed significant differences between the uncoloured Arabic numerals (*M* = 506, *SD* = 113) and colourful Arabic numerals conditions (*M* = 559, *SD* = 93) [*t*_(28)_ = 4.024, *p* < 0.001, *d* = 0.512], the uncoloured Arabic numerals and Chinese numerals conditions (*M* = 682, *SD* = 137) [*t*_(28)_ = 8.377, *p* < 0.001, *d* = 1.701], and the colourful Arabic numerals and Chinese numerals conditions [*t*_(28)_ = 5.249, *p* < 0.001, *d* =1.051] (Figure 2). Additionally, under the carryover condition, the material type had a significant effect [*F*_(2, 56)_ = 48.774, *p* < 0.001, $\eta_{p}^{2}$ = 0.635], and post hoc analysis showed similar significant patterns between uncoloured Arabic numerals (*M* = 523, *SD* = 128) and colourful Arabic numerals (*M* = 575, *SD* = 127) [*t*_(28)_ = 3.180, *p* = 0.004, *d* = 0.408], uncoloured Arabic numerals and Chinese numerals conditions (*M* = 755, *SD* = 149) [*t*_(28)_ = 8.451, *p* < 0.001, *d* =1.670], and colourful Arabic numerals and Chinese numerals conditions [*t*_(28)_ = 6.343, *p* < 0.001, *d* = 1.300] (Figure 2). Regarding the difficulty effect (without carryover vs. with carryover), significance was observed only in the uncoloured Arabic numerals [*t*_(28)_ = 2.150, *p* = 0.040, *d* = 0.141] and Chinese numeral conditions [*t*_(28)_ = 5.852, *p* < 0.001, *d* = 0.510]. In the colourful Arabic numerals condition, the effect of difficulty was not significant [*t*_(28)_ = 1.400, *p* = 0.173, *d* = 0.144] (Figure 2). The sex variable was not the key point we considered in the present study; as a result, we did not analyse sex variables for ERP data.

### 3.2. Analysis of ERP Data

**Figure 3:**
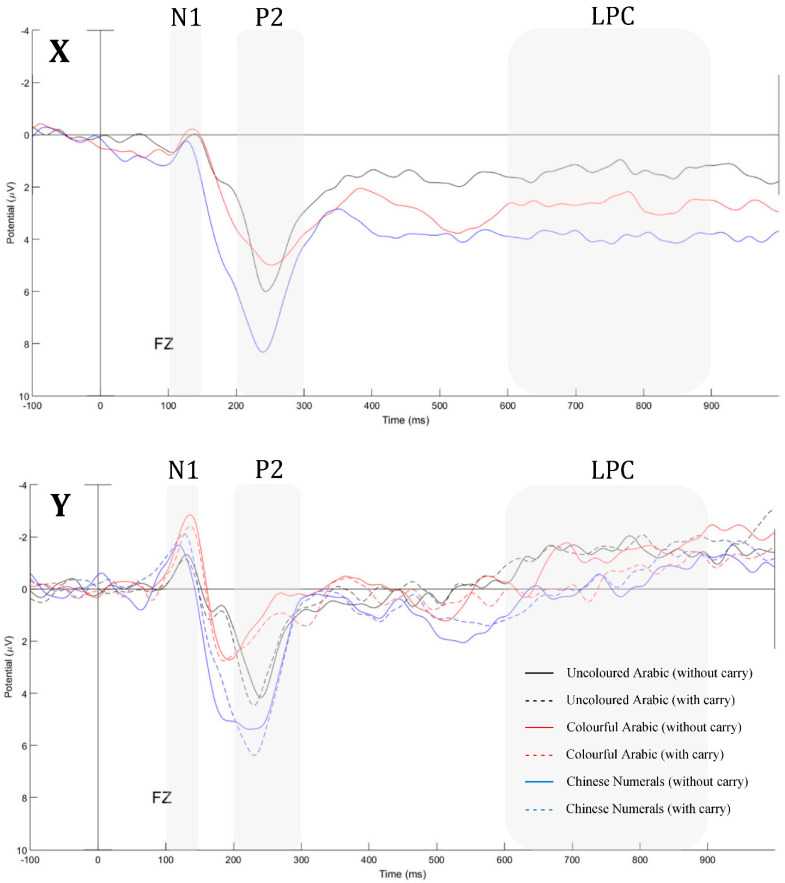
ERP waveforms averaged across participants elicited by the augend number (X) and the addend number (Y) at electrode Fz. Since there is no arithmetic related to the carry operation in the augend step (statistics results support this hypothesis), the without carry and with carry trials were combined in augend number (X).

The ERPs components were analysed by focusing on the first augend number X and the second addend number Y in the simple addition equation (x + y = z). The ERP waveforms evoked by the augend and addend numbers of simple addition equations under the three conditions are presented in Figure 3. Table 2 lists the descriptive statistics (mean and SD) and major statistical results for the mean amplitude (μV) of each ERP component from different time windows. 

Number X (augend);

N1 (100–150 ms):

To examine the effects of material type and difficulty level, a 3 (uncoloured Arabic numerals, colourful Arabic numerals, or Chinese numerals) × 2 (without carryover vs. with carryover) repeated-measures ANOVA was performed on the N1 mean amplitude. However, no significant effect was observed for material category [*F*_(2, 56)_ = 1.151, *p* = 0.324, $\eta_{p}^{2}$ = 0.039], difficulty [*F*_(1, 28)_ < 0.001, *p* = 0.994, $\eta_{p}^{2}$ = 0.000], or the interaction between material category and difficulty [*F*_(2, 56)_ = 0.299, *p* = 0.743, $\eta_{p}^{2}$ = 0.011] (Table 2).

**Figure 4:**
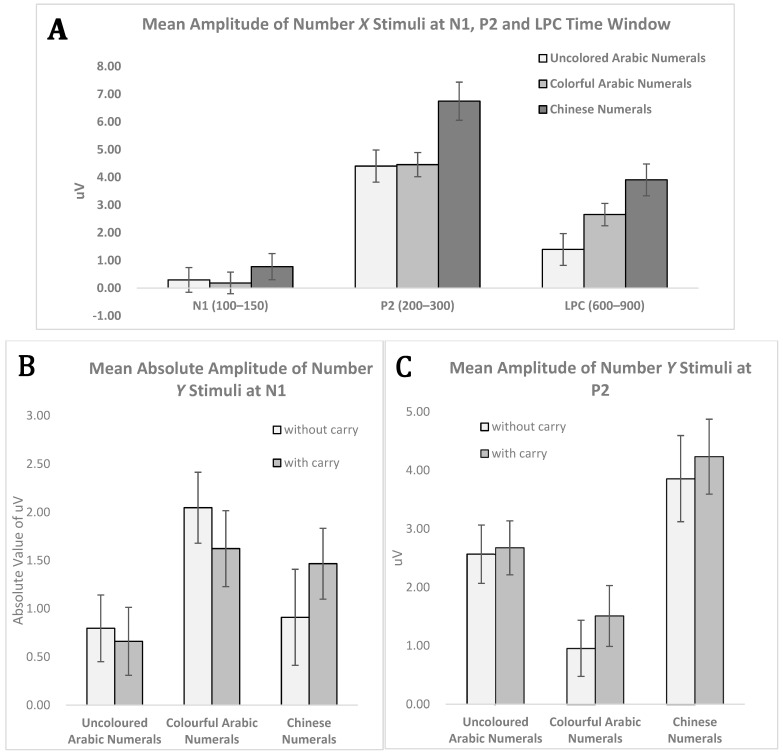
Mean amplitudes of ERP components (error bars indicate standard error). (**A**) Mean amplitude of number *X* stimuli at N1 (100–150 ms), P2 (200–300 ms), and LPC (600–900 ms) Time window (without carryover and with carryover trials are combined here). (**B**) The N1 mean absolute amplitude of number *Y* stimuli under without carryover and with carryover conditions. (**C**) The P2 mean amplitude of number *Y* stimuli under without carryover and with carryover condition.

P2 (200–300 ms): 

The same repeated-measures ANOVA was performed on the P2 mean amplitude. The effect of material type was significant [*F*_(2, 56)_ = 10.382, *p* < 0.001, $\eta_{p}^{2}$ = 0.270], and post hoc analysis revealed that in the Chinese numerals condition (*M* = 6.73, *SD* = 3.64), the P2 value was significantly larger than that in the uncoloured Arabic numerals (*M* = 4.39, *SD* = 3.07) [*t*_(28)_ = 3.898, *p* < 0.001, *d* = 0.695] and colourful Arabic numerals conditions (*M* = 4.45, *SD* = 2.31) [*t*_(28)_ = 3.534, *p* = 0.001, *d* = 0.748]. No significant difference was found between the uncoloured Arabic numerals and colourful Arabic numerals [*t*_(28)_ = 0.123, *p* = 0.903, *d* = 0.022] (Figure 4A). The main effects of difficulty [*F*_(1, 28)_ = 1.343, *p* = 0.256, $\eta_{p}^{2}$ = 0.046] and the interaction between material category and difficulty [*F*_(2, 56)_ = 0.295, *p* = 0.746, $\eta_{p}^{2}$ = 0.010] were not significantly different (Table 2).

LPC (600–900 ms): 

The mean amplitude analysis showed that material type had a significant effect [*F*_(2, 56)_ = 10.310, *p* < 0.001, $\eta_{p}^{2}$ = 0.269] in the three conditions. Further analysis revealed that the LPC mean amplitude in the uncoloured Arabic numerals condition (*M* = 1.40, *SD* = 3.04) was smaller than that in the colourful Arabic numerals condition (*M* = 2.65, *SD* = 2.14) [*t*_(28)_ = 2.796, *p* = 0.009, *d* = 0.476 ] and Chinese numerals condition (*M* = 3.91, *SD* = 3.07) [*t_(_*_28)_ = 4.462, *p* < 0.001, *d* = 0.822]. Further, the colourful Arabic numerals and Chinese numerals conditions exhibited a marginally significant difference [*t*_(28)_ = 1.987, *p* = 0.057, *d* = 0.476], revealing that the Chinese numerals condition caused a larger LPC than the colourful Arabic numerals condition (Figure 4A). The main effect or interaction effect reached significance for the LPC mean amplitude [*F*_(1, 28)_ = 0.914, *p* = 0.347, $\eta_{p}^{2}$ = 0.032], [*F*_(2, 56)_ = 0.538, *p* = 0.587, $\eta_{p}^{2}$ = 0.019].

Number Y (addend)

N1 (100–150 ms): 

To examine the effects of material type and difficulty level, a 3 (uncoloured Arabic numerals, colourful Arabic numerals, Chinese numerals) × 2 (without carryover vs. with carryover) repeated-measures ANOVA was performed on the N1 mean amplitude. The main effect of material type was significant [*F*_(2, 56)_ = 5.266, *p* = 0.008, $\eta_{p}^{2}$ = 0.158], and further analysis revealed that in the colourful Arabic numerals condition (*M* = −1.83, *SD* = 1.72), a larger negative amplitude on N1 was found compared to that in the uncoloured Arabic numerals condition (*M* = −0.73, *SD* = 1.35) [*t*_(28)_ = 3.223, *p* = 0.003, *d* = 0.711]. The main effect of difficulty level [*F*_(1, 28)_ = 0.000, *p* = 0.998, $\eta_{p}^{2}$ < 0.001] and the interaction between material type and difficulty [*F*_(2, 56)_ = 1.281, *p* = 0.286, $\eta_{p}^{2}$ = 0.044] were not found to be significant (Figure 4B).

P2 (200–300 ms):

To examine the effects of material type and difficulty level, a 3 (uncoloured Arabic numerals, colourful Arabic numerals, Chinese numerals) × 2 (without carryover vs. with carryover) repeated-measures ANOVA was performed on the P2 mean amplitude. The main effect of material type was significant [*F*_(2, 56)_ = 19.947, *p* < 0.001, $\eta_{p}^{2}$ = 0.416]. The P2 mean amplitude under the Chinese numerals condition (*M* = 4.05, *SD* = 3.41) was significantly larger than that under the uncoloured Arabic numerals condition (*M* = 2.62, *SD* = 2.04) [*t*_(28)_ = 3.377, *p* = 0.002, *d* = 0.509] and colourful Arabic numerals condition (*M* = 1.23, *SD* = 2.38) [t _(28)_ = 5.519, *p* < 0.001, *d* = 0.959]. The P2 mean amplitude in the uncoloured Arabic numerals condition was significantly larger than that in the colourful Arabic numerals condition [*t*_(28)_ = 3.500, *p* = 0.002, *d* = 0.627]. The main effects of difficulty [*F_(1, 28)_* = 1.550, *p* = 0.223, $\eta_{p}^{2}$ = 0.052] and the interaction between material type and difficulty [*F*_(2, 56)_ = 0.198, *p* = 0.821, $\eta_{p}^{2}$ = 0.007] were not significantly different (Figure 4C).

LPC (600–900 ms): 

No main or interaction effect reached significance on the LPC mean amplitude [*F*_(2, 56)_ = 0.850, *p* = 0.433, $\eta_{p}^{2}$ = 0.029], [*F*_(1, 28)_ = 0.147, *p* = 0.705, $\eta_{p}^{2}$ = 0.005], [*F*_(2, 56)_ = 0.534, *p* = 0.589, $\eta_{p}^{2}$ = 0.019] (Table 2).

N1 and P2 Correlation

Before further correlation analysis, the N1 component values were multiplied by a negative sign to convert their numerical direction from upside-down to upright. As shown in Table 3, we observed that virtually all of the mean amplitudes of the N1 and P2 components in the augend number X and addend number Y from three distinct material categories showed a significant negative association, with the exception of addition with carryover in the Chinese numerals condition. This might provide evidence that N1–P2 processing reacts to the early phases of attention allocation. The significant correlation between N1 and P2 suggests participants’ cognitive plasticity, but cognitive overload due to complex arithmetic operations (such as addition with carryover) in inappropriate symbols (Chinese numerals) may disrupt the N1–P2 relationship.

## 4. Discussion

This study aimed to measure cognitive load and understand ERP components for extraneous and germane cognitive load based on cognitive-load theory [1,11,17]. The study participants performed the weakest in the Chinese numerals condition in terms of accuracy and reaction time (Figure 2) and were consistent with the prediction. As the prediction, reaction time in the colourful Arabic numerals condition was shorter than that in the Chinese numeral conditions [35]. The results indicate that the participants solved the simple addition task in Chinese numerals condition as increasing elements unnecessarily to induce extraneous cognitive load [36]. In other words, extraneous cognitive load may be affected by instructional design, which is not productive for the learner [37]. Chinese numerals, as an extraneous cognitive load, are not an appropriate design to access information for mathematic computations. Additionally, the P2 component exhibited the highest amplitude in the Chinese numerals condition (Figure 3). The P2 component was consistent with the magnitude of cognitive load [38] and may have resulted from low-frequency two-character compound words [30].

Further, in terms of accuracy and reaction time, the participants performed poorly in the addition with carryover condition compared to without the carryover. However, no significant differences were found between the P2 amplitudes with and without carryover conditions for the addend (y number). In other words, operational difficulty may not have affected the P2 amplitude in this task, but the stimuli did affect the P2 amplitude. We speculate that the performance for the difficulty variable may be a floor effect, and we did not observe any differences in this phase of the present study. Another possibility is that the P2 component was related to extraneous cognitive load, rather than difficulty level, and belonged to an inefficient cognitive load for solving problems in this task. This issue requires further study for confirmation.

In the colourful Arabic numerals condition, reaction times and accuracy were not the best among these three conditions for behavioural data, but we noted the largest N1 waveform, which suggests that the participants paid more attention to the colourful numbers because attention to stimuli would induce a larger N1 amplitude [39]. However, a colourful number may enhance performance [40,41].

According to the rationale and results, extraneous and germane cognitive load are very different. However, they may also share a common cognitive structure. Except for carryover calculations in the Chinese numeral condition, the results (Table 3) indicate a negative correlation between N1 and P2, suggesting that the N1 component may have modulated or affected the P2 component. These patterns and behavioural data are similar to those observed by Gao et al. [35]. This is the first study to demonstrate a relationship between germane and extraneous cognitive load. This implies that they may form a common cognitive structure through ERP implementation.

## 5. Concluding Remarks

Based on the studies by Pass et al. [11] and Pass et al. [17], we measured students’ cognitive load during mathematical computation by tracking their brain activity using ERP. Separating the experiment into three conditions (uncoloured Arabic numerals, colourful Arabic numerals, and Chinese numerals), we instructed the participants to perform addition equations and calculations. The Chinese numerals condition was used to assess participants’ extraneous cognitive load; the colourful Arabic numerals condition was used to assess their germane cognitive load; and the uncoloured Arabic numerals condition was used as a control condition. We noted that the largest P2 amplitude was present in the Chinese numerals condition as an extraneous cognitive load, and the N1 component was present in the colourful Arabic numerals condition as a germane cognitive load. For the same mathematical questions, if the numbers are presented in different conditions, differences in brain activity can be found. These findings indicate a pattern for these differences. The largest P2 amplitude may represent an increase in extraneous cognitive load, whereas the N1 component may represent a germane cognitive load. Further, there is association between N1 and P2 amplitudes. It implied that N1 may affect P2 component.

According to the results, brain activity is a suitable parameter for measuring cognitive load [38,40,41]. This study also supports the arguments of Sweller [1] and Mayer [10]. The highest P2 amplitude and N1 component can be used as physiological indicators to assess learners’ cognitive load. In other words, the findings can be applied to brain–computer interfaces.

According to statements proposed by Sweller [1] and Mayer [10], cognitive load has a significant influence on students’ learning effectiveness. If instructional design or teaching material design is poor, it increases the cognitive load of learners in the learning process and influence learning effectiveness. However, according to Pass et al. [11] and Pass et al. [17], at present, the measurement of cognitive load is mainly carried out in postlearning surveys, whereas physiological techniques, such as eye movements and brain waves, can be measured in the process of learning. Nevertheless, this area needs further research. The findings of this study can be applied to the real-time measurement of cognitive load in e-learning. Through the establishment of a portable brain computer interface and real-time algorithm, computers can detect the cognitive load of learners in the learning process and automatically adjust the e-learning environment to improve the cognitive load of learners, so as to improve learning effectiveness. This aspect is worth further investigation.

This study has some limitations. Few studies have examined the relationship between the cognitive-load theory and brain activity. Therefore, there may be an issue regarding the rationale of the experimental design. Second, the participants were university students, and the task involved simple mathematical equations. Other aspects, such as age, remain to be studied. Hence, we suggest that further research should be conducted to investigate participants of different ages, using materials with content from different subjects or fields. Moreover, the visual angles of the materials were not the same across all conditions because the Chinese numerals were wider than the other types used. If the materials in the Chinese numerals condition were manipulated to a size that was the same as that of others, it would be difficult to recognise Chinese characters. Finally, this was a preliminary study rather than an experimental paradigm, and further research is required to construct relevant rationale and details for educational applications.

## Figures and Tables

**Table 1:** The mean percentages of corrected behavioural performance in percentages (hit–false alarm) on simple addition tasks and the correct response time in milliseconds on simple addition tasks. Standard deviations are shown in parentheses.

	Without Carryover			With Carryover		
	Female Hit-FA	Male Hit-FA	All Hit-FA	Female Hit-FA	Male Hit-FA	All Hit-FA
Uncoloured Arabic Numerals	0.96 (0.04)	0.96 (0.05)	0.96 (0.05)	0.91 (0.08)	0.92 (0.08)	0.92 (0.08)
Colourful Arabic Numerals	0.97 (0.05)	0.96 (0.05)	0.97 (0.05)	0.92 (0.11)	0.94 (0.07)	0.93 (0.09)
Chinese Numerals	0.94 (0.06)	0.95 (0.08)	0.94 (0.07)	0.86 (0.07)	0.89 (0.14)	0.88 (0.11)
	Female (Hit) RT Time	Male (Hit) RT Time	All (Hit) RT Time	Female (Hit) RT Time	Male (Hit) RT Time	All (Hit) RT Time
Uncoloured Arabic Numerals	486 (107)	528 (119)	506 (113)	495 (119)	553 (134)	523 (128)
Colourful Arabic Numerals	553 (73)	565 (113)	559 (93)	581 (126)	567 (133)	575 (127)
Chinese Numerals	635 (144)	733 (112)	682 (137)	709 (141)	805 (146)	755 (149)

**Table 2:** The mean amplitudes (μV) of the N1, P2, and LPC components for the augend number (X) and the addend number (Y) stimulus are provided for the three separate material categories (uncoloured Arabic numerals, colourful Arabic numerals, and Chinese numerals) at two different difficulty levels (addition without carryover and addition with carryover). Standard deviations are shown in parentheses.

		Uncoloured Arabic Numerals		Colourful Arabic Numerals		Chinese Numerals		Material Main Effect	Difficulty Main Effect	Material × Difficulty Interaction
		Without Carry	With Carry	Without Carry	With Carry	Without Carry	With Carry			
N1 (100–150)	X	0.14 (2.40)	0.45 (2.70)	0.26 (2.23)	0.12 (2.68)	0.86 (2.59)	0.69 (3.05)	*F*_(2, 56)_ = 1.151, *p* = 0.324,	*F*_(1, 28)_ = 0.000, *p* = 0.998,	*F*_(2, 56)_ = 0.299, *p* = 0.743,
	Y	–0.80 (1.83)	–0.66 (1.87)	–2.05 (1.95)	–1.62 (2.08)	–0.91 (2.63)	–1.47 (1.94)	*F*_(2, 56)_ = 5.266, *p* = 0.008,	*F*_(1, 28)_ = 0.000, *p* = 0.994,	*F*_(2, 56)_ = 1.281, *p* = 0.286,
P2 (200–300)	X	4.44 (3.06)	4.36 (3.41)	4.52 (2.92)	4.39 (2.41)	7.04 (4.02)	6.45 (3.86)	*F*_(2, 56)_ = 10.382, *p* < 0.001	*F_(1, 28)_* = 1.343, *p* = 0.256,	*F*_(2, 56)_ = 0.295, *p* = 0.746
	Y	2.57 (2.64)	2.68 (2.44)	0.96 (2.54)	1.51 (2.75)	3.86 (3.90)	4.23 (3.39)	*F*_(2, 56)_ = 19.947, *p* < 0.001	*F_(1, 28)_* = 1.550, *p* = 0.223,	*F*_(2, 56)_ = 0.198, *p* = 0.821
LPC (600–900)	X	1.72 (3.58)	1.07 (2.95)	2.57 (3.51)	2.74 (2.41)	4.31 (4.31)	3.50 (3.41)	*F*_(2, 56)_ = 10.310, *p* < 0.001	*F*_(1, 28)_ = 0.914, *p* = 0.347	*F*_(2, 56)_ = 0.538, *p* = 0.587
	Y	–1.39 (4.78)	–1.56 (4.57)	–1.21 (3.89)	–0.41 (3.30)	–0.18 (5.25)	–0.34 (5.57)	*F*_(2, 56)_ = 0.850, *p* = 0.433	*F*_(1, 28)_ = 0.147, *p* = 0.705	*F*_(2, 56)_ = 0.534, *p* = 0.589

**Table 3:** Correlations between N1 mean amplitudes and P2 mean amplitudes elicited by the numbers X and Y resulting from different difficulty levels (without carryover or with carryover) for different material categories (uncoloured Arabic numerals, colourful Arabic numerals, and Chinese numerals). It is worth noting that for numerical direction adjustment conversion, the values of the N1 component were multiplied by a negative sign.

Pearson Correlation Alpha	Uncoloured Arabic Numerals		Colourful Arabic Numerals		Chinese Numerals	
	X	Y	X	Y	X	Y
N1–P2 (without carry)	−0.612 **	−0.538 **	−0.688 **	−0.504 **	−0.732 **	−0.729 **
N1–P2 (with carry)	−0.746 **	−0.488 **	−0.525 **	−0.488 **	−0.641 **	−0.226

** *p* < 0.01.

## Data Availability

The data presented in the present study are available upon request of the corresponding author.
